# Progress in State Medicine

**Published:** 1902-05-24

**Authors:** 


### Progress in State Medicine.

**Tuberculosis.**—Brown [1] is of opinion that too much money is spent per bed in consumption sanatoria, and that unless there is a radical change in this respect, the poorer classes will be debarred from treatment. He suggests that where an administrative block has been erected, or a mansion purchased for the treatment of consumption, a series of shelters should be erected at a short distance off, containing from six to twelve beds each; such shelters should not cost more than £20 or £30 per bed. Small dressing-rooms behind would enable patients to dress and wash in privacy. The wash water would run out in a simple filter as described by Poore, and no sewerage would be needful. The bedclothes would be removed and hung up by day, the mattress covered by a sheet, and so a couch provided for reclining on. Elaborate baths would not be provided, but the patient should be taught to get a complete wash with a basin of water, a sponge, and a coarse towel, so that this portion of the treatment shall be continued when he returns home. The patients should be required to make their own beds and keep these shelters clean, do all the gardening work about the place under the direction of the gardener and supervision of the doctor. Any refusing to work would be discharged or compelled to pay the entire cost of their treatment. Gardening is the best work for such sufferers, and many leaving the sanatorium would be able to secure such employment. At present they are only taught to be loafers, and at the recent Congress, work, instead of idleness, was insisted upon by many of the speakers. Robertson [2] thinks the most important problem is how to deal with advanced cases of phthisis among the artisan and generally less wealthy classes. These refuse to enter the parochial hospitals, and usually seek admission into the general hospitals and infirmaries, where they are generally turned away. Consequently they must return to their homes, too often in the most densely populated districts, where overcrowding is common, and where they become centres for the diffusion of tubercle, both in the home circle and around it. It would be blameworthy to admit an advanced consumptive into the general ward of an infirmary or hospital. It is asserted by some that in large well-lighted wards where the sputa is carefully disinfected, and other precautions taken, the introduction of phthisical cases does not constitute a danger to the other inmates, and they point out the lack of evidence that either nurses or patients have ever contracted the disease in this manner. Robertson states, however, that he knows of three nurses, a house physician, and a patient, who there is strong reason to believe acquired phthisis in the wards of a large hospital. He advocates that one or two large wards should be reserved for such cases in hospitals where there is no available land adjoining for building special shelters. Where land is available phthisis wards should be built, preferably of brick, which could be taken down and renewed every few years. The open-air system should be followed both for the good of the patient and the instruction of students. If municipal hospitals were built for the advanced cases, many would refuse to take advantage of them, owing to the gloomy associations of an institution solely devoted to conditions mostly hopeless. Such institutions, too, would be built at some distance from the towns or cities, thus separating the patients a considerable distance from their relatives and friends. No more suitable place away from their homes could be found than the hospital of the town or district in which their homes are situated. There would be fewer removals to their homes arising from the longing to be with their relations when the illness assumes a more serious aspect. Looking at it from another point, the fact that all advanced cases do not die, and that such agents as tuberculin, germicides, and antitoxins, are every day giving hopeful results, it is important that cases of well-marked phthisis should be admitted into our hospitals for scientific study.

In addition these patients have a claim, moral if not legal, on the infirmaries and hospitals, in that they contribute year after year to their support through the workers' fund where they have been employed. The very poor are better off for treatment than the artizan classes. They are accommodated in the parish or union hospitals. In a hospital in course of erection by the Glasgow Parish Council for 1,500 chronic patients of this class, there will be special wards provided for consumptive cases. Attention [3] is drawn to the fact that far too many of our school buildings are most inadequately ventilated and very imperfectly lighted. In these unhealthy rooms children and teachers are compelled to work in an atmosphere which produces headache, lassitude, anæmia, and chronic ill-health, all of which predispose to consumption. Even in many of the schools recently built the subject of ventilation has received far too little attention. Thorough ventilation is a necessity. In addition children and teachers should secure during a portion of each day such an amount of active exercise as will bring about full inflation of the lungs. Expectoration by consumptive pupils must be checked, and every class should be provided with proper material for cleansing their slates. Schoolrooms should be swept some considerable time before the children enter them, the dust being burnt and not thrown on to some neighbouring dust-heap to be blown about the playground. Parents should be advised to deal promptly with children suffering from colds in the head and chest, and to consult a doctor in the case of glandular swellings in any part of the body. A tendency to consumption is in itself no bar to attendance at school. A child suffering from actually developed phthisis ought to stay away as fit only for medical treatment, and as infectious and dangerous to the other scholars.

It is pointed out in the Eleventh Report of the State Board of Health, Maine, U.S.A.[4], that in examining statistics relating to treatment of consumptive patients in sanatoria two facts must be borne in mind, that a large proportion of the patients have not entered these institutions during the incipiency of the disease, when it is most easily and rapidly curable; and that many of them leave the sanatoria after a comparatively short stay. Many of the patients have only been put fairly well on the road to recovery. Weicker's statistics showed that in his sanatorium, intended for the poorer classes of patients, the average stay was only about 10 weeks. Of the 546 persons discharged in the year 1898, 93 per cent. were improved. When leaving the sanatorium, 63 per cent. of them were capable of resuming their work, 15 per cent. were able to engage in light work, and 21 per cent. were unable to resume their work at all. In 1899 a careful inquiry was made into the condition of all the patients who had left the home in the preceding years. Only 8 per cent. were not heard from. Of the 1898 patients, 48 per cent. could work, 18 per cent. were dead; of the 1897 patients, 44 per cent. could work, 30 per cent. were dead; of the 1896, 30 per cent. could work, 50 per cent. were dead; of the 1895, 33 per cent. could work, 50 per cent. were dead. Much more satisfactory were the results with the patients who were in the first stage of the disease upon entry, and who were capable of resuming their work when they left the sanatorium. Of these discharged in 1898, 95 per cent. were still able to work, in 1897, 97 per cent., and in 1896, 100 per cent. were still able to work. Of 68 persons discharged in 1896 from the sanatorium at Grebowsee, in Germany, as fully or partially capable of continuing their former work, 51 per cent. had, up to March 1899, been able to follow their work regularly; and 17 per cent. more had worked most of the time. It has been suggested that there is danger in bringing a large number of consumptives together in a single institution—first the patients endanger each other; secondly, that the people in the surrounding country are endangered. In some open health resorts the visits of large numbers of consumptives have caused a marked increase in the prevalence of consumption among the native population. When, however, the incoming patients have been kept under strict discipline, so far as the care of their sputa is concerned, there have been no evidences of danger to fellow patients and attendants, nor to the inhabitants of the neighbouring districts. A sanatorium for consumptives should be so situated that it may be conveniently reached from the most populous parts — easily accessible from the main railway lines, not on them. An interior location, rather than proximity to the ocean, is preferable. There should be an absence of insect pests which abound in some places at certain seasons of the year. Newman,[5] writing on phthisis in relation to house property in Finsbury, states that in 1900 there were 309 deaths from consumption in that district, a death rate of 2·91 per 1,000. It was an almost universal rule that these deaths occurred in that portion of the district upon which houses are overcrowded on the land without open space, and in houses of comparatively low rental. Closure would seem to be the only remedy, and for insanitary areas a more or less thorough clearance.

This condition of house property has been brought about mainly by two agencies. In the first place, whole streets and squares of houses formerly occupied by single families are now occupied as separate dwellings on separate floors. Sanitary conveniences designed for one family now provide for four. Secondly, the yards in the rear of the houses have, in many cases, been built over, thus effectually preventing through ventilation on the ground floor. The houses have thus become, practically speaking, back-to-back houses. It is noticeable that in somewhat similar properties, but in immediate proximity to open spaces, deaths from phthisis during the last five years have been very few and far between. Out of a total of 1,294 deaths from this disease during the last five years, 18 per cent. occurred in houses in which there were, during the five years, two or more deaths from phthisis. Of such houses there were 116, 95 of which were used for ordinary residential purposes. Yet almost without exception the phthisical patients in these houses neglected to take any precautionary measures whatever with regard to expectoration or disinfection. Experience in Finsbury goes to prove that house infection is due to want of cleanliness and want of fresh air. The main requirements for the prevention of consumption are: (1) voluntary notification, (2) thorough cleansing and disinfection of surfaces, (3) instruction of the public, (4) more cubic space in house property. Morris,[6] speaking at Winchester, stated that the National Association for the Prevention of Consumption had been trying to establish branches in all the big centres of the country. They had been singularly successful in many, but in other towns they had not been able, at present, to arouse even the slightest interest in the subject. There was no danger to any neighbourhood by the establishment of sanatoria, nor was there any danger from individuals in their own houses, provided the rules for protecting others were carried out. Boards of Guardians should combine not only among themseves but with other sanitary authorities, and not to build sanatoria for paupers alone; it would be far better to build sanatoria in which some of the patients could contribute to their own comfort. There should be beds in respect to which other public bodies, for example the friendly societies, might contribute to their maintenance. Lord Salisbury had told them to expect no help from the State. His answer to that was "as soon as the people of this country are really moved by this question, then the Government will do what the people want."

[1] Tuberculosis, Oct. 1901. [2] B. M. J., Feb. 22, 1902. [3] Tuberculosis, Jan. 1902. [4] Sanit. Rec., Feb. 27, 1902. [5] Tuberculosis, Jan. 1902. [6] B. M. J., Mar. 1, 1902.

